# A preliminary study of the association between the *ABCA1* gene promoter DNA methylation and coronary artery disease risk

**DOI:** 10.22099/mbrc.2018.28910.1312

**Published:** 2018-06

**Authors:** Habib Ghaznavi, Khalil Mahmoodi, Mohammad Soleiman Soltanpour

**Affiliations:** 1 Health Promotion Research Centre, School of Medicine, Zahedan University of Medical Sciences, Zahedan, Iran; 2 Department of Cardiology, School of Medicine, Zanjan University of Medical Sciences, Zanjan, Iran; 3 Department of Medical Laboratory Sciences, School of Paramedical Sciences, Zanjan University of Medical Sciences, Zanjan, Iran

**Keywords:** Coronary artery disease, Methylation, ATP binding cassette transporter A1

## Abstract

Coronary artery disease (CAD) is a common health problem in Iranian population. ATP binding cassette transporter A1 (ABCA1) plays central role in the efflux of the cholesterol from peripheral tissues back to liver. Inactivation of ABCA1 by epigenetic change such as DNA methylation may contribute to the development of CAD. The present study investigated the association between promoter DNA methylation status of *ABCA1* with the development and severity of CAD. Our study population consisted of 110 angiographically documented CAD patients and 110 controls. The severity of CAD was determined based on the number of stenotic vessels showing more than 50% stenosis. Promoter DNA methylation status of *ABCA1* was determined by methylation specific PCR. Lipid profile was determined by routine colorimetric methods. Results showed that the frequency of *ABCA1* DNA methylation was significantly higher in CAD group as compared with control group (16.36% *vs* 5.45%; P=0.015). Also, the methylation frequency of *ABCA1* gene was significantly higher in older CAD patients as compared with younger CAD patients (P=0.038). No association was seen between plasma lipid concentration and the promoter DNA methylation status of *ABCA1* (P>0.05). Also, the association between the severity of CAD and methylation of *ABCA1* gene was not significant (P>0.05). In conclusion the current study indicated *ABCA1* DNA methylation as a significant risk factor for development but not severity of CAD. Also, predisposition to the development of CAD by *ABCA1* gene DNA methylation was independent of plasma lipid concentration.

## INTRODUCTION

Coronary artery disease (CAD) is a common and preventable health problem in many developed and developing countries. During the past years, the prevalence of CAD has been increased in Iran [1]. Several mechanisms including defects in inflammatory response signaling pathways and abnormalities in the lipid metabolism pathways have been involved in the pathogenesis of coronary heart disease [2, 3]. Excessive accumulation of cholesterol loaded macrophages, known as foam cells, may play pivotal roles in the formation and development of atherosclerotic lesions. 

The formation of foam cells in the arterial intima is the result of imbalance between influx and efflux of cholesterol and has been considered as a pathological hallmark of CAD [4]. Several key regulators are involved in the cholesterol traffic across the cell membranes including ATP binding cassette transporter A1 (ABCA1) and G1 (ABCG1) which are the members of ATB transporter superfamily [4]. The ABCA1 transporter participates in initial phase of reverse cholesterol transportation (RCT) by effluxing of the cholesterol from peripheral cells and foam cells to lipid-poor apolipoprotein AI and thereby creating nascent high-density lipoprotein particles (HDL) [5]. However, a study, demonstrated that Monocyte/macrophage ABCA1 contributed to HDL formation, but the contribution to the overall plasma HDL levels was minimal [6]. The defect in the ABCA1 function may impair the RCT activity and could result in the formation of lipid loaded macrophages and foam cells in arterial walls [5, 7]. 

The *ABCA1* resides on chromosome 9q31.1 and several mutations have been identified in this gene [8, 9]. Mutations in the *ABCA1* were shown to be involved in the pathogenesis of Tangier disease characterized by lack of HDL-C in plasma and an increased tendency to develop premature cardiovascular disease [10]. Pervious studies have indicated a link between mutations or single nucleotide polymorphisms in the *ABCA1* and CAD risk [9, 11, 12]. In addition to genetic changes, epigenetic alteration such as DNA methylation associated with gene inactivation may contribute to the risk of coronary heart disease [13-15]. DNA methylation, the most common and stable epigenetic change, is characterized by the addition of methyl group to CpG dinucleotide in the promoter of gene, that generally cause suppression of gene expression [15, 16]. The association between epigenetic DNA methylation alteration and coronary heart disease or atherosclerosis has been investigated in several studies [17, 18]. The role of promoter DNA methylation status of *ABCA1* in the pathogenesis of CAD has been addressed in a few studies [13, 14]. So, because of the critical function of ABCA1 transporter in the regulation of lipid metabolism and RCT function, the present study investigated the association between *ABCA1* promoter DNA methylation status with the development and severity of CAD risk and also with the lipid profile in an Iranian sub-population.

## MATERIALS AND METHODS

**Study subjects:** The investigated population consisted of 110 angiographically documented CAD patients (56 men and 54 female) admitted for angiography center of Moussavi Medical and Educational Center of Zanjan and 110 ethnically matched healthy subjects (55 men and 55 female) as control. All CAD patients were inspected with an expert cardiovascular specialist and were included in the study based on a minimum stenosis of 50% in at least one major coronary vessel on angiography. The severity of CAD was determined according to the number of stenotic vessels showing more than 50% stenosis and thereby patients were categorized into single-, double- and triple vessel stenosis. The CAD patients showing luminal stenosis of <50% or taking lipid-lowering drugs were not included in the study. Also, patients with infectious disease, inflammatory disease, auto immune disorders and major organ failure were excluded from the study. Control subjects with a family history of CAD or in the presence of a concomitant disease such as auto immune disease, febrile disease, malignant diseases or organ failure were excluded from the study. Information regarding the smoking habits, hypertension (as defined by systolic blood pressure>140 mmHg and/or diastolic blood pressure >90 mmHg) and diabetes (as defined by fasting blood glucose >126mg/dL), family history of heart disease, hyperlipidemia and the presence of any acute or chronic disease was obtained from all participants. The study was approved by the ethical committee of Zanjan University of Medical Sciences (Ethical code: ZUMS.REC.1394.268). Written informed consent was obtained from all of the study subjects.

**Blood sample collection and Biochemical assays:** After 12 hours fasting, 5 ml blood was collected in EDTA containing tubes and immediately was centrifuged. The plasma fraction was used for biochemical measurements and the cellular fraction was used for DNA extraction. The plasma levels of biochemical markers including glucose, triglyceride (TG), total cholesterol (TC), high density lipoprotein cholesterol (HDL-C) and low density lipoprotein cholesterol (LDL-C) were assayed using calorimetric methods in a Mindray auto-analyzer (BS-200) according to standard protocols provided by commercial enzyme assay kits (Pars Azmoon Co, Tehran, Iran). 

**Methylation analysis:** Genomic DNA was isolated from blood leukocytes using a commercially available DNA extraction kit (Viogene, Poland) according to the manufacturer’s protocol. By using a commercially available EpiTect Bisulfite Kit (Qiagen, Germany), approximately 2 µg of extracted DNA was treated by bisulphite sodium and subsequently was eluted by 50 µl elution buffer and stored at -20 ºC until analysis. 

The promoter DNA methylation status of *ABCA1* was determined by methylation specific PCR (MSP). Two set of primers specific for methylated and un-methylated status of DNA promoter region were designed using the MethPrimer/ tools and Databases/The Li Lab (http://www.urogene.org/methprimer/). The sequence of primers used for MSP of ABCA1 gene were as follows: (methylated F: AAT TTT ATT GGT GTT TTT GGT TGT C, methylated R: ATA TCT TAA AAT CCG CGA TCT ACG and (un-methylated F: AAT TTT ATT GGT GTT TTT GGT TGT T, un-methylated R: TAT CTT AAA ATC CAC AAT CTA CAT C). Hot Start Master Mix (Ampliqon, Denmark) was used for amplification of target gene in a standard PCR protocol using 10 µm of each primer with an annealing temperature of 58ºC for methylated and 60ºC for un-methylated state. Appropriate positive controls (EpiTect PCR Control DNA Set, Qiagen, Germany) were used in each PCR reaction. Following completion of PCR reaction, the amplified products were electrophoresed on a 3% agarose gel. The size of PCR products with methylated and un-methylated - primers were 139bp and 138bp, respectively. 

**Statistical methods:** The numerical data presented as mean ± SD and were compared using Student t-test. The categorical variables presented as absolute and percentage and were compared using Chi square test or Fisher's exact tests. Also, binary Logistic regression analysis was used to detect the independent association of each risk factor with CAD. The statistical analyses were done using the SPSS 16 statistical software (SPSS Inc., Chicago, Ill) and a P-value less than 0.05 was considered statistically significant.

## RESULTS

The investigation of demographic, clinical and laboratory features of study population revealed no significant differences regarding the mean age and sex distribution between the two groups (P>0.05). However, significant differences was seen regarding the plasma triglyceride, total cholesterol, HDL-C, LDL-C, diabetes, hypertension and smoking habits between the two studied group. 

Next, we evaluated the frequency of *ABCA1* DNA methylation in study population. Statistical analysis using Fisher's exact test indicated that the presence of *ABCA1* DNA methylation was significantly more common in CAD group than control group [18 out of 110 CAD patients (16.36%) *vs* 06 out of 110 controls (5.45%), OR=3.39; 95% CI=1.29-8.90; P=0.015]. Moreover, investigating the prevalence of *ABCA1* DNA methylation between the older (age>65 years, mean age: 74.7±8.2; n=38) and younger (age<65 years, mean age: 53.9±9.9; n=72) CAD patients indicated that 11 out of 38 older CAD patients had methylated ABCA1 gene, while 7 out of 72 younger CAD patients revealed methylated *ABCA1* gene. Statistical analysis using Fisher's exact test indicated that the frequency of DNA methylation was significantly more common in CAD patients with old age relative to CAD patients with younger age (OR=2.97; 95% CI: 1.06-8.30, P=0.038). However, when the frequency of DNA methylation were compared separately between the two groups of CAD patients and the control group, significant differences was only seen between the older CAD patients and the control group (P=0.001). 

In order to investigate the independent association of each variable with the risk of CAD, multiple binary logistic regression analysis was done. Considering the study group (CAD group vs. control group) as the dependent variable and age, sex, TG, TC, HDL-C, LDL-C, smoking, diabetes, hypertension and *ABCA1* methylation status as covariates, the methylated state of *ABCA1* was identified as a significant and independent risk factor for CAD development (OR=2.68, 95% CI: 1.12-7.13; P=0.028). Moreover, other variables such as smoking (P=0.008), hypertension (P=0.027), TG (P=0.006), TC (P<0.001), HDL-C (P<0.001) and LDL-C (P=0.028) displayed a significant and independent effect on CAD risk. However, some other variable including age (P=0.406), sex (P=0.951) and diabetes (P=0.072) were not identified as significant covariate in the regression analysis. Next, we investigated the correlation between *ABCA1* DNA methylation and lipid profile. As, presented in Table 1, no significant differences was seen regarding the plasma lipid levels between the methylated and un-methylated status of *ABCA1 *in both CAD group (P>0.05) and control group (P>0.05). 

**Table 1:** The association between lipid concentrations with *ABCA1* DNA methylation status

**Methylation status**	**TG**	**TC**	**HDL-C**	**LDL-C**
**CAD group**				
**Un-methylated**	194.39±81.42	206.23±62.14	39.35±8.42	103.93±34.19
**Methylated**	190.06±90.38	195.87±52.6	35.98±8.36	88.90±42.02
***P***	0.840	0.509	0.124	0.104
**Control group**				
**Un-methylated**	162.15±67.29	168.75±47.23	46.48±12.99	91.98±33.02
**Methylated**	190.06±90.38	181.51±103.07	43.76±8.53	82.03±29.17
**P**	0.508	0.576	0.614	0.472

The interaction between CAD and some acquired risk factors such as diabetes, hypertension and smoking on the DNA methylation of *ABCA1* was evaluated. Results indicated only significant impact of smoking on the DNA methylation of *ABCA1* in CAD cases (Table 2). However, because of low number of DNA methylation in control group, the effect of these risk factors on DNA methylation was ignored in controls. 

**Table 2:** **:** Interaction between coronary artery disease and some acquired risk factors on the *ABCA1* DNA methylation status.

	**Methylated (n=18)**	**Un-methylated (n=92)**	**OR (95% CI)** *	***P*** **-value ** *
**Diabetes**	09	19	2.42 (0.94-6.20)	0.106
**Hypertension**	06	20	1.53 (0.54-4.35)	0.403
**Smoking**	12	22	2.78 (1.17-6.62)	0.028

* Calculated by Fisher's exact test.

The association between the severity of CAD, as determined by the number of stenotic vessels showing more than 50% stenosis and the methylation status of ABCA1 gene was also investigated. Results indicated no significant differences regarding the frequency of ABCA1 methylation between CAD patients with single, double and triple stenotic vessels (P>0.05) (Table 3).

**Table 3:** The frequency of *ABCA1* DNA methylation in CAD patients with the single, double and triple stenotic coronary vessels

**Methylation state**	**1 SV** **n=39**	**2 SV** **n=46**	**3 SV** **n=25**	**2SV ** ***vs*** ** 1SV**	**3SV ** ***vs*** ** 1SV**
				**P** *	**P** *
**Un-methylated**	34 (87.12)	40 (86.96)	18 (72.0)	Ref	Ref
**Methylated**	05 (12.82)	06 (13.04)	07 (28.0)	0.993	0.189

* Calculated using Fisher's exact test.

## DISCUSSION

The present study investigated the role of *ABCA1* DNA methylation in the development and severity of CAD in an Iranian subpopulation. ABCA1 is a key regulator of revers cholesterol transport from peripheral tissues back to liver [19]. Previous studies have shown that genetic variations in the *ABCA1* gene may contribute to the development of CAD [9,11,20]. The present study indicated promoter DNA methylation of *ABCA1* as a significant risk factor for development but not for severity of CAD. Similarly, in a recently published study by Guay et al. the *ABCA1* DNA methylation was reported as a significant and independent risk factor for CAD [21]. Also, in another study in the patients with familial hypercholesterolemia it was indicated that *ABCA1* gene promoter methylation was associated with CAD occurrence [14]. Peng et al. in a study of 85 coronary heart diseases (CHD) has reported that *ABCG1* gene DNA methylation was more common among CHD patients relative to CHD free subjects [22]. Our preliminary results indicated *ABCA1 *gene promoter methylation as a significant risk factor for CAD occurrence in older CAD patients but not in younger age ones. Similarly, other studies have also reported significant association between *ABCA1 *gene promoter methylation and aging [21]. The higher *ABCA1* DNA methylation levels observed in CAD patients with old age could also be related to a longer disease history that can lead to accumulation of epigenetic changes in these patients [23].

The current study found no significant association between *ABCA1 *promoter methylation and plasma lipid concentrations. This result is inconsistent with some previous studies [13, 14]. However, in a mice model that was selectively deficient in leukocyte *ABCA1* locus, significant increase in the CAD occurrence was seen without considerable change in the plasma HDL-C levels [24]. It should be noted that alteration in the *ABCA1* could affect RCT activity and the net flux of cholesterol from the vessel wall toward the liver, and thereby may influence the development and or severity of CAD without necessarily modifying plasma lipid levels [7, 11, 25]. Our study was also in agreement with the study of Peng et al. that found no significant association between *ABCG1* methylation and the lipid profile in a recently published study [22]. In the present study, a significant effect of smoking on DNA methylation was seen in CAD patients. So, *ABCA1* DNA methylation may be contributed to the interaction of CAD and smoking. This finding may provide an epigenetic mechanism by which tobacco smoking increases the risk of CAD. Similarly, some previous studies have demonstrated the effect of tobacco smoking on DNA methylation of CAD-related genes [26, 27]. Some limitations exist in the present study including (i) the gene expression levels of *ABCA1* gene was not investigated (ii) the assay of the cellular cholesterol efflux activity which is a better indicator of ABCA1 activity was not evaluated. 

## Conflict of Interest:

There is no conflict of interests to be declared regarding the publication of this paper.

## References

[1] Sarrafzadegan N, Sadeghi M, Oveisgharan S, Iranipour R (2013). Incidence of cardiovascular diseases in an Iranian population: the Isfahan Cohort Study. Arch Iran Med.

[2] Seidi A, Mirzaahmadi S, Mahmoodi K, Soltanpour MS (2018). The association between NFKB1-94ATTG ins/del and NFKB1A -826C/T genetic variations and coronary artery disease risk. Mol Biol Res Commun.

[3] Nelson RH (2013). Hyperlipidemia as a risk factor for cardiovascular disease. Prim Care.

[4] Yu XH, Fu YC, Zhang DW, Yin K, Tang CK (2013). Foam cells in atherosclerosis. Clin Chim Acta.

[5] Annema W, Tietge UJ (2012). Regulation of reverse cholesterol transport-a comprehensive appraisal of available animal studies. Nutr Metab (Lond).

[6] Haghpassand M, Bourassa PA, Francone OL, Aiello RJ (2001). Monocyte/macrophage expression of ABCA1 has minimal contribution to plasma HDL levels. J Clin Invest.

[7] Wang MD, Franklin V, Marcel YL (2007). In vivo reverse cholesterol transport from macrophages lacking ABCA1 expression is impaired. Arterioscler Thromb Vasc Biol.

[8] Brunham LR, Singaraja RR, Hayden MR (2006). Variations on a gene: rare and common variants in ABCA1 and their impact on HDL cholesterol levels and atherosclerosis. Annu Rev Nutr.

[9] Lu Y, Liu Y, Li Y, Zhang H, Yu M, Kanu JS, Qiao Y, Tang Y, Zhen Q, Cheng Y (2015). Association of ATP-binding cassette transporter A1 gene polymorphisms with plasma lipid variability and coronary heart disease risk. Int J Clin Exp Pathol.

[10] Puntoni M, Sbrana F, Bigazzi F, Sampietro T (2012). Tangier disease: epidemiology, pathophysiology, and management. Am J Cardiovasc Drugs.

[11] Ghaznavi H, Aali E, Soltanpour MS (2018). Association study of the ATP - binding cassette transporter A1 (ABCA1) rs2230806 genetic variation with lipid profile and coronary artery disease risk in an Iranian population. Open Access Maced J Med Sci.

[12] Cyrus C, Vatte C, Al-Nafie A, Chathoth S, Al-Ali R, Al-Shehri A, Akhtar MS, Almansori M, Al-Muhanna F, Keating B, Al-Ali A (2016). The impact of common polymorphisms in CETP and ABCA1 genes with the risk of coronary artery disease in Saudi Arabians. Hum Genomics.

[13] Guay SP, Brisson D, Lamarche B, Gaudet D, Bouchard L (2014). Epipolymorphisms within lipoprotein genes contribute independently to plasma lipid levels in familial hypercholest-erolemia. Epigenetics.

[14] Guay SP, Brisson D, Munger J, Lamarche B, Gaudet D, Bouchard L (2012). ABCA1 gene promoter DNA methylation is associated with HDL particle profile and coronary artery disease in familial hypercholesterolemia. Epigenetics.

[15] Sayols-Baixeras S, Irvin MR, Elosua R, Arnett DK, Aslibekyan SW (2016). Epigenetics of lipid phenotypes. Curr Cardiovasc Risk Rep.

[16] Lim DH, Maher ER (2010). DNA methylation: a form of epigenetic control of gene expression. Obstet Gynecol.

[17] Fernández-Sanlés A, Sayols-Baixeras S, Subirana I, Degano IR, Elosua R (2017). Association between DNA methylation and coronary heart disease or other atherosclerotic events: A systematic review. Atherosclerosis.

[18] Hai Z, Zuo W (2016). Aberrant DNA methylation in the pathogenesis of atherosclerosis. Clin Chim Acta.

[19] Wang X, Collins HL, Ranalletta M, Fuki IV, Billheimer JT, Rothblat GH, Tall AR, Rader DJ (2007). Macrophage ABCA1 and ABCG1, but not SR-BI, promote macrophage reverse cholesterol transport in vivo. J Clin Invest.

[20] Qi LP, Chen LF, Dang AM, Li LY, Fang Q, Yan XW (2015). Association between the ABCA1-565C/T gene promoter polymorphism and coronary heart disease severity and cholesterol efflux in the Chinese Han population. Genet Test Mol Biomarkers.

[21] Guay SP, Légaré C, Houde AA, Mathieu P, Bossé Y, Bouchard L (2014). Acetylsalicylic acid, aging and coronary artery disease are associated with ABCA1 DNA methylation in men. Clin Epigenetics.

[22] Peng P, Wang L, Yang X, Huang X, Ba Y, Chen X, Guo J, Lian J, Zhou J (2014). A preliminary study of the relationship between promoter methylation of the ABCG1, GALNT2 and HMGCR genes and coronary heart disease. PloS one.

[23] Rowbotham DA, Marshall EA, Vucic EA, Kennett JY, Lam WL, Martinez VD (2015). Epigenetic changes in aging and Age-related disease. J Aging Sci.

[24] Van Eck M, Bos IS, Kaminski WE, Orso E, Rothe G, Twisk J, Böttcher A, Van Amersfoort ES, Christiansen-Weber TA, Fung-Leung WP, Van Berkel TJ (2002). Leukocyte ABCA1 controls susceptibility to atherosclerosis and macrophage recruitment into tissues. Proc Natl Acad Sci USA.

[25] Kyriakou T, Hodgkinson C, Pontefract DE, Iyengar S, Howell WM, Wong YK, Eriksson P, Ye S (2005). Genotypic effect of the-565C>T polymorphism in the ABCA1 gene promoter on ABCA1 expression and severity of atherosclerosis. Arterioscler Thromb Vasc Biol.

[26] Zeilinger S, Kühnel B, Klopp N, Baurecht H, Kleinschmidt A, Gieger C, Weidinger S, Lattka E, Adamski J, Peters A, Strauch K (2013). Tobacco smoking leads to extensive genome-wide changes in DNA methylation. PLoS One.

[27] Steenaard RV, Ligthart S, Stolk L, Peters MJ, van Meurs JB, Uitterlinden AG, Hofman A, Franco OH, Dehghan A (2015). Tobacco smoking is associated with methylation of genes related to coronary artery disease. Clin Epigenetics.

